# Small retained foreign bodies: what is the limit of detection using current emergency ultrasound equipment?

**DOI:** 10.1186/2036-7902-4-S1-A12

**Published:** 2012-12-18

**Authors:** Daniel Jafari, KJ Cody, NL Panebianco, FS Shofer, BS Ku, A Au, AJ Dean

**Affiliations:** 1 Department of Emergency Medicine, Hospital of the University of Pennsylvania, Philadelphia, PA, USA; 2 Department of Emergency Medicine, Thomas Jefferson University Hospital, Philadelphia, PA, USA

## Background

Previous studies of small foreign bodies (FB) have shown a wide range of accuracies of FB detection using animal models, with high accuracy rates for FB > 10 mm and variable accuracy rates for 4 to 5 mm FB.

## Objective

To determine the lower limit of sonographic detection of FB using current emergency ultrasound equipment in a soft tissue model.

## Methods

FBs made of metal, glass, wood, and plastic (3 of each) 1 x 1 x 3 mm in size were placed at a depth of 0.5-2.0 cm in 12 pork feet. 8 feet were punctured without FB placement. Pork feet were submerged during this process to minimize air in tissue. 7 ED sonologists with > 2 years experience were blinded to overall number, type and depth of FB, but not to size. FB sites were scanned by each sonologist using either a hockey stick or traditional linear array transducer in a randomized pre-assigned order. Sonologist confidence in the diagnosis was reported using a visual analog scale for each site. Sensitivity, specificity, positive and negative predictive values (PPV and NPV) with 95% confidence intervals were calculated. To determine if sonologist confidence differed by perceived presence or absence of a foreign body, paired t-test was used.

## Results

140 ultrasound scans were performed which reported sensitivity, specificity, PPV and NPV as 50% (95% CI: 39%-61%), 50% (37%-61%), 60% (48%-72%), and 40% (28%-52%) respectively. There was little agreement among the sonologists (only 2 sites with 100% agreement). Sensitivity ranged from 25% to 75%, specificity 37% to 62%, PPV 42% to 75%, and NPV 25% to 57% for each sonologist. Sonologists were more confident reporting a positive result (81% vs 51%, p<0.0001), irrespective of the actual presence of FB. The difference between detection rates of 4 types of FB did not reach statistical significance.

## Conclusion

Current emergency ultrasound equipment utilized by ED sonologists is unreliable in detection of 3 mm FB in a human extremity soft tissue model. Future studies may further delineate accuracy rates among different sizes and materials of FB.

